# Characterizing Selected Sorghum Grain Varieties and Evaluating the Suitability of Their Malt Extracts for Cultivating Microbial Biomass

**DOI:** 10.1155/2021/6658358

**Published:** 2021-12-17

**Authors:** Stellah Byakika, Ivan Muzira Mukisa, Yusuf Byenkya Byaruhanga

**Affiliations:** Department of Food Technology and Nutrition, School of Food Technology Nutrition and Bioengineering, College of Agricultural and Environmental Sciences, Makerere University, P.O. Box 7062 Kampala, Uganda

## Abstract

Microbial biomass is cultivated for different technological applications including food processing, medicine, waste management, and research. The conventional growth media used are generally expensive thus necessitating the development of more affordable alternatives. In this study, four sorghum grain varieties, SESO 1, SESO 3, *Epuripur*, and *Eyera*, and their malt extracts were characterized which is aimed at determining their suitability for growing microbial biomass. The varieties had kernel length, kernel width, kernel thickness, and thousand kernel weigh equivalent to 3.8-4.3 mm, 3.2-4.5 mm, 2.4-2.8 mm, and 12.4-20.2 g, respectively. SESO 1 and *Epuripur* had corneous endosperm textures whereas those of SESO 3 and *Eyera* were intermediate and floury, respectively. Varieties had germinative energies > 90% and total defects < 8%. SESO 3 had the highest (*p* < 0.05) crude protein (10.8 ± 0.3%) and dietary fiber (22.5 ± 0.4%) whereas *Epuripur* had the highest (*p* < 0.05) starch (81.6 ± 0.0%) and crude fat (2.9 ± 0.1%). There was no significant difference (*p* > 0.05) in the ash contents (2.1 ± 0.0%). The total sugars, free amino nitrogen, condensed tannins, and pH of the malt extracts were 106-116 g/L, 70-78 mg/L, 0.1-0.6 mg/mL, and 5.5-5.7, respectively. The composition of the sorghum malt extracts suggests their potential for use in cultivating microbial biomass.

## 1. Introduction

Sorghum is a staple cereal whose cultivation is key in improving household nutrition, food security, and incomes, especially in developing countries. Consequently, breeding programs are continuously developing varieties that are high yielding, early maturing, and resistant to disease, pests, and drought. In Uganda, the National Semiarid Resources Research Institute (NaSARRI) developed sorghum varieties, SESO 1, SESO 3, and *Epuripur*, that are high yielding and disease tolerant [1]. *Epuripur* and SESO 1 are low in tannins and suitable for lager beer brewing. SESO 3 has a high concentration of tannins and is thus more suitable for food and local brewing [1]. Malted grain is of particular interest in food processing because of its better nutritional profile and lower antinutrient content compared to unmalted grain [2, 3]. Hence, it is popular in brewing and infant food formulations.

Traditional nonalcoholic fermentation processes and several researches have demonstrated that sorghum malt on its own can support the growth of microorganisms like lactic acid bacteria [4–7]. It is, therefore, possible that sorghum malt could potentially be a good growth medium for microbial biomass. Some groups of microorganisms such as lactic acid bacteria (LAB) have fastidious nutritional requirements and are inhibited by antinutrients [8, 9]. Therefore, a sorghum malt product may support their luxurious growth.

Microbial biomass has a range of biotechnological applications including fermentation of foods, probiotics, production of pharmaceuticals, and waste treatment among others. Several bacteria and fungi are vital in the fermentation of cereals, fruits, honey, vegetables, legumes, fish, and meats imparting characteristic flavors and tastes while contributing to food product safety and preservation [10]. Some strains belonging to *Bifidobacterium breve*, *Bifidobacterium lactis*, *Bifidobacterium infantis*, *Bacteroides fragilis*, *Lactobacillus*, *Escherichia coli* and *Faecalibacterium prausnitzii*, and *Saccharomyces boulardii* are used as probiotics owing to their ability to impart health benefits on their hosts upon consumption of adequate amounts [11]. Microorganisms are also used to produce a range of pharmaceutical products including antibiotics, antifungal agents, hormones, and enzymes [12]. Microbes are also applied in decomposition and decontamination of various types of wastes such as liquid and solid refuse [13].

Microbial biomass is generally first propagated to large concentrations prior to its successful application in biotechnological processes. Propagation is carried out in conventional synthetic growth media which are expensive and not readily available especially in developing countries [14–16]. This necessitates identification of cheaper alternatives which should be rich in sugars (preferably simple sugars), a nitrogen source, minerals (especially manganese and magnesium), and B complex vitamins [8, 15]. Sorghum grains are rich in starch and also contain proteins and micronutrients [17]. Since malting increases nutrient bioavailability and lowers antinutrients, sorghum malt extract is potentially a good propagation medium for microbial biomass. This work, therefore, characterized selected sorghum varieties and their malt extracts to determine their suitability as propagation media for microbial biomass.

## 2. Materials and Methods

### 2.1. Sorghum Varieties

Four sorghum varieties (SESO 1, SESO 3, *Epuripur*, and *Eyera)* were used in this study. SESO 1 and *Epuripur* are white grained whereas SESO 3 is brown grained. *Eyera* is a popular local brown grained variety [18]. All four varieties were obtained from NaSARRI in Serere district, Uganda. The grains were assayed for defects, germinative energy, thousand kernel weight, kernel size, endosperm texture, and proximate composition prior to use in making sorghum malt extracts.

### 2.2. Sorghum Malt Extracts

Sorghum grain was malted following procedures described by Taylor [19]. The malted grain was milled using a Wonder Mill (110 Volt model, California, USA) and sieved using a 800 *μ*m screen. The flour was mixed with water to form a mixture of 11% total solids. To convert starch to maltose, the mixture was heated to 75°C, followed by addition of *α*-amylase (Anke Bio Engineering Company Limited, China) at a rate of 1000 units per milliliter. The slurry was held at 75°C for 1 h with continuous stirring. To convert maltose to glucose, the slurry temperature was lowered to 55°C and amyloglucosidase (Anke Bio Engineering Company Limited, China) was added at a rate of 1000 units per milliliter. The slurry was held at 55°C for 1 h with continuous stirring. The malt extract was cooled to about 25°C, decanted, and filtered using grade filter papers (Whatman No. 1). It was then sterilized at 121°C for 15 min and cooled to 25°C. The malt extracts were assayed for free amino nitrogen (FAN), total sugars, pH, and condensed tannins.

## 3. Analyses

### 3.1. Grain Defects

Total defects were determined according to Taylor and Taylor [20]. Twenty-five grams of sorghum grain was weighed in duplicate and spread on an A4 sheet of paper into a monolayer. Using a ruler, all defects were separated out of the good grain, collected, and weighed. Defects were presented as a percentage of the original weight.

### 3.2. Germinative Energy

Germinative energy was determined according to the method described by the European Brewery Convention [21]. A 9 cm diameter filter paper (Whatman No. 1) was placed in a 10 cm diameter glass Petri dish and moistened with 4 mL of distilled water. One hundred intact sorghum grains were spread evenly over the surface of the moistened filter paper in such a way that none of the grains touched each other. The Petri dishes were closed and incubated at 25°C, and the grains were examined after 24, 48, and 72 h. At each time interval, the germinated grains were counted and removed from the Petri dishes. Germinative energy was computed as the percentage of original grains that germinated by 72 h.

### 3.3. Thousand Kernel Weight (TKW) and Kernel Sizes

The TKW was determined by weighing 1000 randomly selected sorghum grains of each variety using an analytical balance (ASB-220-C2-V2, MRC, Germany). Kernel length, width, and thickness of 100 randomly selected sorghum grains of each variety were measured using a vernier caliper (Series 530, Mitutoyo, USA).

### 3.4. Endosperm Texture

**Figure 1:**
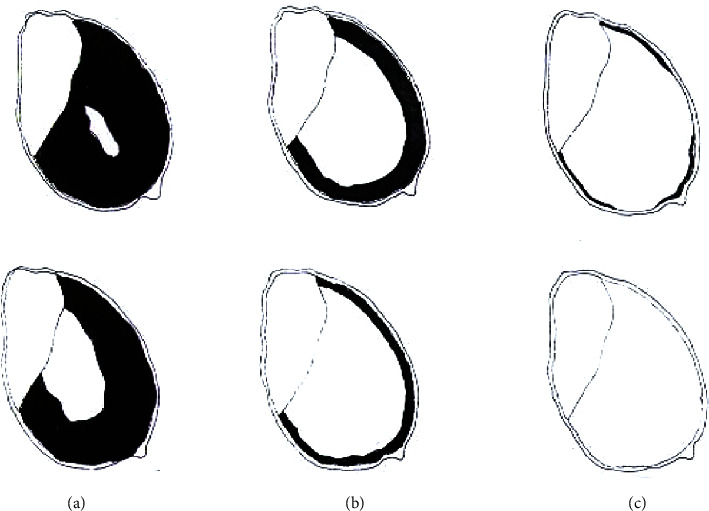
Illustration of sorghum grain endosperm texture. (a) Corneous; (b) intermediate; (c) floury. The corneous endosperm is totally or >50% translucent endosperm. The intermediate endosperm has a continuous outer corneous endosperm comprising <50% of total endosperm while the inner endosperm is floury. The floury endosperm is totally floury or its outer corneous endosperm is very narrow and incomplete (adapted from Taylor and Taylor [22]).

Endosperm texture was determined according to Taylor and Taylor [22]. A small piece of gum was placed onto a piece of paper. A sound sorghum grain with the germ side up was pushed into the side of the gum to hold it in place. The grain was held using a pair of forceps and cut lengthwise into two even size halves. Each half of the grain was compared against Figure 1. The procedure was done for 20 grains of each variety.

### 3.5. Proximate Composition

Proximate composition (starch, crude fat, crude protein, ash, and dietary fiber) of the grains was determined using the Association of Official Analytical Chemists [23]. For the crude protein determination, the nitrogen value obtained was multiplied by a factor of 5.65.

### 3.6. Analysis of Sorghum Malt Extract

Free amino nitrogen (FAN) was determined using the ninhydrin method [21], and the total sugars were determined using the phenol-sulfuric acid method [24]. The pH was measured using a pH meter (AG model, Mettler-Toledo Group, Switzerland). Condensed tannins were determined according to the vanillin method as described by Broadhurst and Jones [25]. Briefly, the condensed tannins were extracted by weighing 0.2 mL of malt extract were transferred to a test tube and mixed with 10 mL of 70% acetone. The mixture was shaken in a water-ice bath for 10 min and subsequently centrifuged for 15 min (1200 g at 4°C). The supernatant was transferred into another test tube and kept on ice away from sunlight. From the supernatant, 40 *μ*l was transferred into another test tube and made up to 250 *μ*L with 50% methanol. For the standard curve, 10 to 70 *μ*l of catechin containing 0.5 mg/mL of 50% methanol was measured and made up to 250 *μ*L using 100% methanol. Finally, 1.5 mL of freshly prepared 4% vanillin was added to the test tube followed by 750 *μ*L of concentrated HCl. The tubes were shaken and left to stand for 10 min. Absorbance was read at 500 nm against using a spectrophotometer (Genesys 10 UV model, Thermo Electron Corporation, USA).

### 3.7. Statistical Analyses

Results were presented as means ± standard deviations (mean ± SD) of three independent experiments. Data were subjected to one-way analysis of variance (ANOVA) to test for significant differences at *α* = 0.05. Mean comparisons were made using the Least Significant Difference (LSD) test. Analyses were done using the Statistix (student version 9.0) software.

## 4. Results and Discussion

### 4.1. Grain Physical Properties

Table 1 summarizes the physical properties of the sorghum varieties. Total defects ranged from 4.3 to 6.7% and were highest (*p* < 0.05) in *Eyera* followed by SESO 3, SESO 1, and *Epuripur*. The germinative energies at 72 h of all varieties were above 90%. There were differences (*p* < 0.05) in kernel sizes and TKW amongst varieties. SESO 1 and *Epuripur* had corneous endosperms while those of SESO 3 and *Eyera* were intermediate and floury, respectively.

Grain defects were applied to all components of a sample which differ from the normal including extraneous matter, insects, blemished, diseased, and broken grains among others [20]. The presence of insects reduces the nutritional value of the grain by depleting the nutrient reserves [26]. Microorganisms, particularly fungi, make grain unpalatable and also contaminate it with mycotoxins [26]. The percentage defects (Table 1) were below 8% which is the maximum stated by Codex Standard 172-1989. This could be because the grain was obtained from NaSARRI a breeding institute where quality control measures are taken to ensure that the grain is properly handled.

Germinative energy is defined as the percentage of grains that will germinate under optimal conditions for the species [27]. The germination energies of the grains at 72 h were similar to those reported by Ogu et al. [28]. Sorghum grain for malting should have a germinative energy at 72 h of ≥90% [20], a recommendation that all varieties studied met (Table 1). In sorghum malt production, it is necessary that a high proportion of grains in a batch germinate so as to activate the different enzyme systems [29]. However, the enzymatic power of malted grain varies with malting conditions and grain variety [30]. With respect to varieties, of the four varieties evaluated, SESO 3 and *Epuripur* had higher germinative energies and would also be expected to have higher enzymatic power.

The TKW and kernel sizes (length, width, and thickness) of the four varieties in this study were similar to those previously reported [31]. Sorghum kernel size and shape are known to affect malting properties including water uptake and germination energy [32]. Variation in kernel sizes is attributed to differences in cultivars [32].

Endosperm texture or hardness is influenced by the proportion of corneous (vitreous or hard) fraction of endosperm with respect to the floury (soft) endosperm [33]. SESO 1, SESO 3, and *Epuripur* would be preferred for malting because corneous and intermediate endosperm textured grains have better water uptake which in turn influences diastatic enzyme activity [32]. This might, therefore, translate into a higher amount of totals sugars as was seen for SESO 1 and *Epuripur* (Tables 2 and 3). A higher concentration of sugars is expected to promote growth of microbial biomass. Consequently, SESO 1 and *Epuripur* might promote better growth of microbial biomass than SESO 3 and *Eyera.* Grain hardness also plays a defensive role against molds and insect attack which would otherwise lower grain viability and nutritional content [33]. The same author reported endosperm texture to also influence milling performance. These authors stated that during decortications, sorghum grains with corneous endosperm textures produce more full endosperms and fewer broken grains than those with floury ones. Kebakile et al. [34] observed that sorghum grains with a floury endosperm generate a higher flour yield and finer particle size on milling compared to those with corneous endosperm. Endosperm texture variations among sorghum varieties are mainly attributed to genetic differences; other factors include environment, moisture, proteins, lipids, and endosperm cell wall [33].

### 4.2. Proximate Composition

Table 2 summarizes the proximate composition of the sorghum grains varieties. SESO 3 had the highest crude protein content (*p* < 0.05) while *Epuripur* had the highest starch and crude fat contents. Dietary fiber was highest in SESO 3 followed by *Epuripur*, SESO 1, and *Eyera* (*p* < 0.05). There were no (*p* > 0.05) varietal differences in ash contents.

The crude protein content of the four varieties agrees with Kigozi et al. [31]. However, the starch content was generally slightly higher (Table 2) than values (65.4-76.3%) reported by Yan et al. [35] and Ragaee et al. [36] which difference could be attributed to varietal differences. Starch is the major component of sorghum grain, constituting about 70% dry grain weight followed by protein [35]. The crude fat and ash values were in agreement with values reported by Ragaee et al. [36] and Yan et al. [35]. It is vital that the high lipid content of *Epuripur* is taken into consideration during processing and storage. Sorghum lipids are highly unsaturated with oleic and linoleic acids accounting for about 80% of the total fatty acids, and these favor lipolysis results in low flour quality [37]. It is, therefore, important that the processing and storage conditions for sorghum minimize rancidity. The dietary fiber contents (Table 2) are close to the value of 21% reported by Ragaee et al. [36]. However, Malleshi et al. [38] reported a lower value (8%) possibly due to differences in varieties and environmental conditions. The nutritional content of sorghum grains is mainly genetically determined although environment factors also play a role [39].

### 4.3. Physicochemical Properties and Nutritional Composition of Sorghum Malt Extract

The FAN, total sugars, condensed tannins, pH, and total soluble solids of the sorghum malt extracts are presented in Table 3. There were differences (*p* < 0.05) in the quantities of total sugars and FAN among the sorghum varieties. The condensed tannins were generally very low, and the pH ranged from 5.5 to 5.7.

FAN comprises the amino acids and peptides produced by proteolytic action of endogenous proteinase and peptidase enzymes on grain protein reserves during malting [40, 41]. According to Mugode [42], sorghum malt extract is relatively rich in FAN. This is attributed to steeping, a process in malting which significantly increases its FAN content [40, 43]. Malting sorghum grain increases proteinase activity significantly [43]. During steeping, moisture content of grain increases to 37.1% activating the enzymes which hydrolyze the grain food reserves during germination [3]. FAN levels of 42-358 mg/L have been reported [28, 42]. The wide range could be attributed to differences in malting conditions particularly the durations of steeping and germination and to a less extent, the sorghum variety [28, 44]. Steeping and germination times have been reported to significantly affect proteolytic activities in sorghum grains [41]. It was observed that steeping and germination time have a great positive influence on proteolytic activity of sorghum varieties, with the optimum steeping and germination times being 40-45 h and 5 days, respectively [41]. In this study, however, shorter steeping and germination times as described in the malting protocol by Taylor [19] were used. The lower FAN levels observed in this study could, therefore, be attributed to the fact that our grain was steeped for only 16 h and germinated for 2-3 days.

FAN is an essential component of yeast nutrition because it promotes proper yeast growth and fermentation efficiency. In brewing for instance, it is recommended that wort should contain about 130 mg/L FAN for proper yeast growth [45]. This requirement could be similar for bacteria; however, amounts as low as 51 mg/L FAN are reported to efficiently support microbial growth [46]. Therefore, 70-78 mg/L FAN obtained in this study could support growth of microbial biomass but it might be necessary to supplement the FAN when growing yeasts. FAN in sorghum can be increased by adding meat peptones, casein peptones, soy peptones, yeast extract, nitrates, and ammonium salts [47].

Dicko et al. [48] reported that the activation of different endogenous amylolytic enzymes during steeping increases the total sugars. Saccharification of the malt extract using commercial exogenous *α*-amylase and amyloglucosidase further contributes to the increase in total sugars. Sorghum grains are predominantly starchy containing up to 70% starch [17]. This explains the high levels of total sugars in the sorghum malt extracts. The differences in total sugars recorded in the sorghum varieties may be attributed to differences in the amount of starch available for hydrolysis. Sugars, in particular glucose, are an important energy source for microbial growth. Microbiological growth media contains 0.25-40 g/L glucose depending on the target microorganisms [47]. The commonest conventional growth medium for lactic acid bacteria (MRS agar or broth) and common media for yeasts and molds (Yeast Chloramphenicol agar and Potato dextrose agar) contain 20 g/L glucose as the major sugar. The malt extract in this work contained about four times more sugar than 20 g/L. Therefore, the presence of high amounts of total sugars in the sorghum malt extracts makes them suitable for microbial biomass growth.

The sorghum malt extracts contained tannins in amounts that were much lower than the maximum (1 mg/mL) allowable for growth of *Lactobacillus* spp. [49, 50]. Tannins inhibit microbial growth mainly by binding to proteins and thus inhibiting enzymes and altering cell membrane permeability [51, 52]. Malting is one of the most common and practical ways of reducing sorghum tannins [3]. For instance, Ojha et al. [53] observed up to 16% reduction in tannins in malted sorghum, so this possibly accounts for the low tannin contents in this study. Of course this is in addition to the dilution effect due to the water added during the saccharification process. The low tannin content observed in the sorghum malt extracts evaluated indicates that these malts are suitable for growth of microbial biomass.

The pH values of the sorghum malt extracts fall within 5.4-8.1, the range of pH for most microbiological growth medium [47]. The values also fall in the range (pH 5.5-6.2) suitable for maximal growth of lactobacilli [9]. Therefore, the pH values of the malt extracts can generally support the growth of a wide range of microorganisms.

## 5. Conclusion

This work is the first of its kind to characterize selected sorghum varieties (SESO 1, SESO 3, *Epuripur*, and *Eyera*) and their malt extracts with the goal of determining their suitability as low-cost propagation media for microbial biomass. The four sorghum varieties and their malt extracts had significant differences in physicochemical properties and composition. *Epuripur* generally had superior qualities. Malt extracts from the four sorghum varieties possessed characteristics (high FAN and total sugars and low condensed tannins and pH) that could support cultivation of microbial biomass. These sorghum varieties could thus potentially be used as low-cost media for propagating microbial biomass. Future studies should focus on *in vitro* evaluation of sorghum extracts and optimizing their composition for growth of specific microorganisms.

## Figures and Tables

**Table 1:** Physical properties of grain sorghum varieties.

Variety	%defects	%GE	TKW (g)	KL (mm)	KW (mm)	KT (mm)	Endosperm texture
SESO 1	4.3^b^ ± 1.5	91.3^bc^ ± 0.6	13.3^c^ ± 0.1	3.9^b^ ± 0.0	3.7^b^ ± 0.1	2.7^a^ ± 0.0	Corneous
SESO 3	5.3^ab^ ± 0.6	94.3^ab^ ± 1.2	13.9^b^ ± 0.2	3.8^c^ ± 0.0	3.2^c^ ± 0.2	2.4^b^ ± 0.1	Intermediate
*Epuripur*	4.3^b^ ± 0.6	94.7^a^ ± 1.5	20.2^a^ ± 0.1	4.3^a^ ± 0.0	4.2^a^ ± 0.0	2.7^a^ ± 0.1	Corneous
*Eyera*	6.7^a^ ± 0.6	90.3^c^ ± 1.5	12.4^d^ ± 0.1	3.3^d^ ± 0.0	3.4^bc^ ± 0.0	2.6^a^ ± 0.0	Floury

GE: germinative energy; TKW: thousand kernel weight; KL: kernel length; KW: kernel width; KT: kernel thickness. Values are means of triplicate determinations ± standard deviations. Mean values in the same column with same superscripts are not significantly different (*p* > 0.05).

**Table 2:** Proximate composition of grain sorghum varieties.

Variety	% starch	% crude protein	% crude fat	% ash	% dietary fiber
SESO 1	78.8^b^ ± 0.5	10.0^b^ ± 0.2	2.4^b^ ± 0.1	2.1^a^ ± 0.0	20.1^c^ ± 0.1
SESO 3	77.4^c^ ± 0.2	10.8^a^ ± 0.3	2.4^b^ ± 0.1	2.1^a^ ± 0.1	22.5^a^ ± 0.4
*Epuripur*	81.6^a^ ± 0.2	9.9^b^ ± 0.0	2.9^a^ ± 0.1	2.1^a^ ± 0.0	21.2^b^ ± 0.2
*Eyera*	76.4^c^ ± 0.1	9.6^b^ ± 0.2	2.4^b^ ± 0.1	2.1^a^ ± 0.0	18.5^d^ ± 0.6

Values are means of triplicate determinations ± standard deviation. Mean values in the same column with same superscripts are not significantly different (*p* > 0.05). Values are on dry matter basis. Nitrogen-to-protein conversion factor was 5.65.

**Table 3:** Composition and physicochemical properties of sorghum malt extracts.

Variety	Total sugars (g/L)	Free amino nitrogen (mg/L)	Condensed tannins (mg/mL)	pH
SESO 1	115.0^b^ ± 0.6	70.0^c^ ± 0.6	0.004^c^ ± 0.1	5.5^c^ ± 0.0
SESO 3	108.0^c^ ± 0.6	74.0^b^ ± 0.6	0.047^b^ ± 0.0	5.6^b^ ± 0.0
*Epuripur*	116.0^a^ ± 1.0	78.0^a^ ± 0.6	0.002^d^ ± 0.1	5.6^b^ ± 0.0
*Eyera*	106.0^d^ ± 0.6	70.0^c^ ± 1.0	0.051^a^ ± 0.0	5.7^a^ ± 0.0

Values are means of triplicate determinations ± standard deviations. Mean values in the same column with the same superscripts are not significantly different (*p* ≥ 0.05).

## Data Availability

The data in tables and figure used to support the findings of this study are included within the article.
